# When do models of NeuroAIDS faithfully imitate “the real thing”?

**DOI:** 10.1007/s13365-017-0601-5

**Published:** 2017-12-18

**Authors:** Benjamin B. Gelman, Janice Endsley, Dennis Kolson

**Affiliations:** 1 Department of Pathology, Route 0419, University of Texas Medical Branch, Galveston, TX 77555-0419 USA; 2 Department of Microbiology and Immunology, University of Texas Medical Branch, Galveston, TX 77555 USA; 3 Department of Neurology, University of Pennsylvania, Philadelphia, PA 19104-6140 USA

**Keywords:** ART, CNS, Dementia, Eradication, HAND, HIV, Latency

## Abstract

HIV-infected patients treated with antiretroviral medicines (ART) still face neurological challenges. HIV-associated neurocognitive disturbances (HAND) can occur, and latent viral DNA persisting in the central nervous system (CNS) prevents eradication of HIV. This communication focuses on how to develop experimental models of HAND and CNS HIV latency that best imitate the CNS pathophysiology in diseased humans, which we take to be “the real thing.” Models of HIV encephalitis (HIVE) with active CNS viral replication were developed in the early years of the AIDS pandemic. The clinical relevancy of such models is in sharp decline because HIVE seldom occurs in virally suppressed patients, while HAND remains common. The search for improved models of HAND should incorporate the neurochemical, neuroimmunological and neuropathological features of virally suppressed patients. Common anomalies in these patients as established in autopsy brain specimens include brain endothelial cell activation and neurochemical imbalances of synaptic transmission; classical neurodegeneration may not be as crucial. With regard to latent HIV with viral suppression, human brain specimens show that the pool of latent proviral HIV DNA in the CNS is relatively small relative to the total body pool and does not change substantially over years. The CNS pool of latent virus probably differs from lymphoid tissues, because the mononuclear phagocyte system sustains productive infection (versus lymphocytes). These and yet-to-be discovered aspects of the human CNS of virally suppressed patients need to be better defined and addressed in experimental models. To maintain clinical relevancy, models of HAND and viral latency should faithfully emulate “the real thing.”

## Establishing models for investigating HAND and CNS viral latency

The pressing need to develop animal models of HIV-associated neurocognitive disorders (HAND) has led to the development of multiple competing models that markedly differ in relevance to an evolving disease state such as HAND. The dictionary defines the word model as “something to be used as an example to imitate or follow another thing.” The model is a surrogate: that which the model seeks to imitate is “the real thing” (i.e., the HAND patient). The focus of this paper and the symposium topic is that the NeuroAIDS field needs to reflect on which biological models are best suited to address the research priorities of the day. We face two clinically relevant research priorities in our time that, if addressed effectively, could lead to specific therapies to treat CNS morbidity in HIV-infected people. One goal is to understand the pathophysiology of CNS dysfunction when HIV replication is suppressed medically with combinatorial antiretroviral therapy (ART), which could lead eventually to better clinical management of HIV-associated neurocognitive disorders (HAND). A second goal is to understand the basic biology of the “latent” pool of HIV DNA in the CNS, which persists in cART-treated patients and prevents virus eradication and cure of HIV/AIDS (Chun and Fauci 1999; Grey et al. 2014; Stevenson, 2017).Understanding the extent to which our biological models emulate the pathophysiology of these two aspects of HIV/AIDS presents a critical challenge in the NeuroAIDS field. The value of our investigation depends upon the clinical relevancy of the experimental systems that we choose to employ. This paper offers no judgment pertaining to the value of one or another experimental model. Those choices will be decided by a field of investigation that is informed about, and focused on, the pathophysiology of “the real thing.”

## Models of HAND in virally suppressed versus non-suppressed patients

A major problem with studying the HAND patient is that HAND is a pathophysiological disease entity that is continuously evolving; its clinical nosology needed to be modified substantially at least once (Antinori et al. 2007). The “old” concept of the pathophysiology of HAND was formulated before the era of ART when viral replication was not suppressed. Patients under observation were younger adults lacking age-related comorbidities. Basic tenets of HAND pathophysiology in the pre-cART era emphasized CNS HIV replication, effects of viral toxins, and neuroinflammation, with resultant neuronal death and dropout (neurodegeneration). Those theoretical concepts, and specifically the notion that HAND is a neurodegenerative disease, reached the status of dogma in routine scientific discourse. It followed naturally that the medical research enterprise would focus attention on lowering viral replication in CNS, controlling brain inflammation, and ultimately protecting neurons from becoming necrotic in predominantly young adults. The initial concepts and biological models of HAND have been based primarily upon the cardinal neuropathological features observed in brain specimens from patients with HIV encephalitis (HIVE). It was believed that eliminating or blunting HIVE and preventing CNS HIV replication could prevent HAND. It was a reasonable hypothesis because animal models of HIVE substantially satisfied modified Koch’s postulates for HIVE but perhaps not HAND (Wiley and Achim 1994). We know now that HIVE nearly never develops in patients given ART, while HAND has remained prevalent. This situation represents both a “neuropathological gap” and a “virological gap” with the clinical diagnosis of HAND in virally suppressed patients (McArthur et al. 2010; Gelman et al. 2013). In virtually all virally suppressed patients, HIVE is not a requirement for HAND and is not the neuropathological substrate. Much of the available information on the neuropathophysiology of HAND, however, still comes from unsuppressed young adult patients with HIVE and/or from animal treatments designed specifically to emulate HIVE. Studies of tissues from virally suppressed, older patients with HAND (without HIVE) more accurately represent patients treated in clinics today. As the persistence of HAND in the absence of HIVE takes hold, new and sharply modified concepts need to emerge, which in turn, requires a re-evaluation of models for their fidelity in mimicking these concepts. Models of CNS viral latency/reservoirs also should emulate what we observe in CNS samples of virally suppressed patients, with and without HAND (Gelman 2015).

## HAND and HIV RNA and DNA in human brain specimens

Before ART, the driving force for HIV-associated dementia (HAD), the severest form of HAND, was CNS HIV replication as represented by the CSF viral load, which was taken to represent HIV replication in cell reservoirs within the brain parenchyma. However, the CSF viral load likely represents a poor surrogate for brain parenchymal viral load (Ellis et al. 1997). Autopsy data on brain HIV from clinically characterized patients did not emerge until the epidemic was well under way. In the largest clinically correlated data set done to date, HIV RNA and DNA were measured in 140 infected people from the National NeuroAIDS Tissue Consortium (NNTC) cohort (Gelman et al. 2013). The data revealed important associations between the HIV RNA pool (which includes replicating virus), the HIV DNA pool (which includes replication competent and incompetent provirus), and HAND. As expected, HIVE patients had higher levels of HIV RNA (copies/gram tissue), likely representing replicating virus. Virus replication at the highest levels (> 10^4^ c/g) was indeed correlated with HAND. Less expected was that viral replication at or below those levels was not correlated significantly with HAND. Further, HAND patients without HIVE did not have higher CNS HIV replication relative to people without HAND, suggesting no requirement for heightened HIV replication and expression of HAND dysfunction. Results from NNTC autopsy brains agree with clinical studies which show that HAND is diagnosed in patients with sustained systemic and CSF viral suppression, and who would not likely have HIVE (Heaton et al. 2010; Heaton et al. 2015). Gene expression data on NNTC autopsy brains showed that *CD163* mRNA, which marks the macrophages that support HIV replication in HIVE brains (Kim et al. 2006), is not significantly higher in virally suppressed patients with HAND versus without HAND (Gelman et al. 2012a). Correlations between worse neuropsychological test performance and brain HIV RNA levels were significant in the autopsy studies only if the unsuppressed patients with high levels of CNS viral replication and HIVE were included in the computations.

It is tempting to speculate that the residual (“latent”) HIV DNA in the CNS is what continues to drive HAND in virally suppressed patients. Preliminary results from brain HIV DNA assays do not support that suggestion. Brain HIV DNA levels showed a lack of significant correlation with neuropsychological test performance in virally suppressed patients (Gelman et al. 2013). Assay for integrated HIV DNA in the brain (versus total HIV DNA), which is more likely to represent truly latent virus, produced the same essential outcome in 29 patients. In total, the available autopsy data suggest that brain viral load (replicating virus) drives HAND in unsuppressed patients with HIVE but not in virally suppressed patients. Correlations between HAND and HIV DNA are weaker generally than for HIV RNA in virally suppressed and unsuppressed patients both. Observations in clinically well-characterized decedents have implications regarding what should, or should not, be imitated in models of virally suppressed patients with HAND.

## Potential inflammatory biomarkers of HAND in human specimens

If the concentrations of detectable viral replication or “latent” HIV DNA do not drive HAND in virally suppressed patients, what should our models seek to imitate? One widely suggested possibility is ongoing CNS inflammation (“neuroinflammation”), leading eventually to neurodegeneration that persists due to smoldering infection (Carroll and Brew 2017; Chen et al. 2014; Manji et al. 2013; Spudich et al. 2011). Residual inflammation could in turn trigger a wide variety of changes systemically including damaging effects to neurons in patients with HAND (Ellis et al. 2007). Clinically, we can observe evidence of transient HIV replication in the plasma of suppressed patients as “viral blips,” which are transient increases in the levels of HIV RNA, suggesting transient escape from ART suppression (Nettles et al. 2005; Lee et al. 2006). In the CNS compartment, the prevalence of “CSF viral escape” is estimated to be 6 to 21% (Rawson et al. 2012; Kugathasan et al. 2017; Mukerji et al. 2017; Eden et al. 2010).One study did not find a direct association between CSF blips and CSF markers of neuronal injury but did find an association with increased expression of neopterin, a marker of macrophage activation (Edén et al. 2016). The potential role for CSF viral blipping in promoting recurrent, transient neuroinflammation and potential neurological dysfunction is nearly impossible to establish in a cross-sectional autopsy survey and will rely strongly on the use of models.

Some clinical observations are compatible with an “early hit” to the CNS in HIV infection, with resulting damage and limited progression thereafter during viral suppression. Longitudinal neuroimaging data suggest that loss of brain volume measurements is a limited, early manifestation of HIV entry into and replication within the CNS, prior to effective viral suppression with ART, and that progressive neurodegeneration thereafter is unusual unless comorbid effects of aging increase the damage to the vulnerable brain. Clinical data show that up to 70% of HAND patients do not clinically progress on suppressive ART (Heaton et al. 2015; Saylor et al. 2016). Neuroimaging shows consistent, non-progressive, regional brain volume reductions in HIV-infected virally suppressed patients (Sanford et al. 2017). Brain volume loss apparently occurs in HIV-infected patients during the first year of infection (Ragin et al. 2015; Wright et al. 2016). The temporal sequences observed using brain imaging are highly important aspects of the overall HAND scenario, and they cannot be confirmed in a cross-sectional autopsy survey.

The issues discussed above suggest that candidate biomarkers should include inflammatory mediators and markers of neurodegeneration. The search for practical biomarkers in HAND patients, using either clinic CSF specimens or neurochemical study at autopsy, has not produced a workhorse biomarker to diagnose or follow HAND in the clinic. Some markers have, however, been correlated with the clinical diagnosis of HAND (Carroll and Brew 2017). A few CSF biomarkers of HAND might be useful for longitudinal tracking of inflammation (neopterin) (Kamat et al. 2012). Markers in blood plasma that reflect systemic whole body reactions to HIV infection have potential utility. Significantly correlated markers of HAND often reflect macrophage activity in blood plasma (soluble CD14 and CD163 and gut-derived lipopolysaccharide) or reflect a systemic change such as the correlation between HAND and the anemia of chronic inflammation (Ancuta et al. 2008; Clifford and Ances 2013; Kallianpur et al. 2016; Carroll and Brew 2017). These inflammatory type biomarkers are easy to emulate in models that feature unrestrained viral replication and HIVE-like changes in the CNS. Whether or not the markers are reproduced faithfully in models of HAND that imitate virally suppressed patients remains to be elucidated. The use of novel functional neuroimaging as a “biomarker” in HAND offers some promise for assessing neuroinflammation in HIV-infected patients on suppressive cART in research settings, because one can emulate a histological evaluation of microglia/macrophage activation within the brain in living patients (Vera et al. 2017).

## Relevancy of markers of neurodegeneration in human samples

Protecting against neurodegeneration (neuroprotection) in HAND has been a frontline therapeutic goal for some years, and neuroprotection is still a major feature of current research output (Ellis et al. 2007). With regard to what markers of neurodegeneration might be desirable to have in a model of HAND in virally suppressed patients, CSF and plasma neurofilament assays are commonly used in HAND clinic studies and HAND models (Abdulle et al. 2007; Beck et al. 2015a, b; Gisslén et al. 2015). A counterpoint to the conventional neurodegeneration-based clinical narrative is that HAND in virally suppressed patients no longer fits the pathological picture of a neurodegenerative disease (Gelman 2015). Embracing this argument triggers a major dialog shift regarding the priority of our therapeutic targeting. This matter enjoys lively and active debate currently in symposia such as this one. The evidence (or lack of) for neurodegeneration in HAND is reflected in the results of brain gene arrays. A brain specimen that contains neurodegeneration should contain a loss of neuronal transcripts, or at the least some dysregulation of the neuronal transcriptome in response to the damage. When over 54,000 brain transcripts were examined in virally suppressed patients with HAND (*n* = 6) and without it (*n* = 6), neuronal transcripts were hardly found to be altered in virally suppressed patients without HIVE (Gelman et al. 2012a, b). To date, no neurodegenerative pathology such as Alzheimer’s (AD), Parkinson’s (PD), or motor neuron disease (ALS) that has undergone similar scrutiny of the brain transcriptome has revealed such little evidence of altered regulation of neuronal gene transcription. This distinguishes classically progressive neurodegenerative diseases such as AD, PD, and motor neuron disease (MND) from HAND, which appears to be non-progressive in up to 70% of virally suppressed patients (Saylor et al. 2016). It also is possible that structural damage to synapses and dendrites in virally suppressed patients without HIVE that occurs is not lethal, which would be compatible with the lack of neuronal dropout that appears at autopsy (Ellis et al. 2007). If that were true, one would expect neuronal gene expression to be abnormal in response to the structural changes in the synaptodendritic arbor, but that is not what brain array data have revealed. To portray synaptodendritic damage graphically, one must select examples from unsuppressed patients with replicating virus and HIVE. Extrapolating these images to virally suppressed patients can incorrectly imply that the two types of HAND patients have one pathophysiology. After over 20 years of ART in clinical practice, it remains unclear if there is a definable underlying neuropathological hallmark of HAND in the absence of HIVE.

## HAND and neurovascular biology in human samples

If we choose to design a model that avoids dependence on neurodegeneration and viral replication both, as discussed above, what aspect is deemed to be worth imitating? Human brain chemistry and systemic changes measured in blood plasma offer some intriguing openings to explore. In the brain, gene array data suggested a broad-based endothelial activation in the brain of six virally suppressed patients with HAND (Gelman et al. 2012a). Confirmatory neurochemical data from 449 HIV-infected brain specimens showed that at least three established endothelial cell gene transcripts (*PECAM1*, *VWF*, and *TFRC*) are higher in HIV-infected people (Buzhdygan et al. 2016). Sampling of blood plasma also shows that a systemic endothelial disturbance occurs in virally suppressed clinic patients (de Gaetano et al. 2004). Since endothelial cells are in constant and dynamic contact with blood plasma, a disturbance transmitted from blood plasma by brain microvascular endothelial cells is compatible with the studies showing that HAND is associated with plasma inflammatory markers and could be driven systemically (Wolf et al. 2002).

**Fig. 1:**
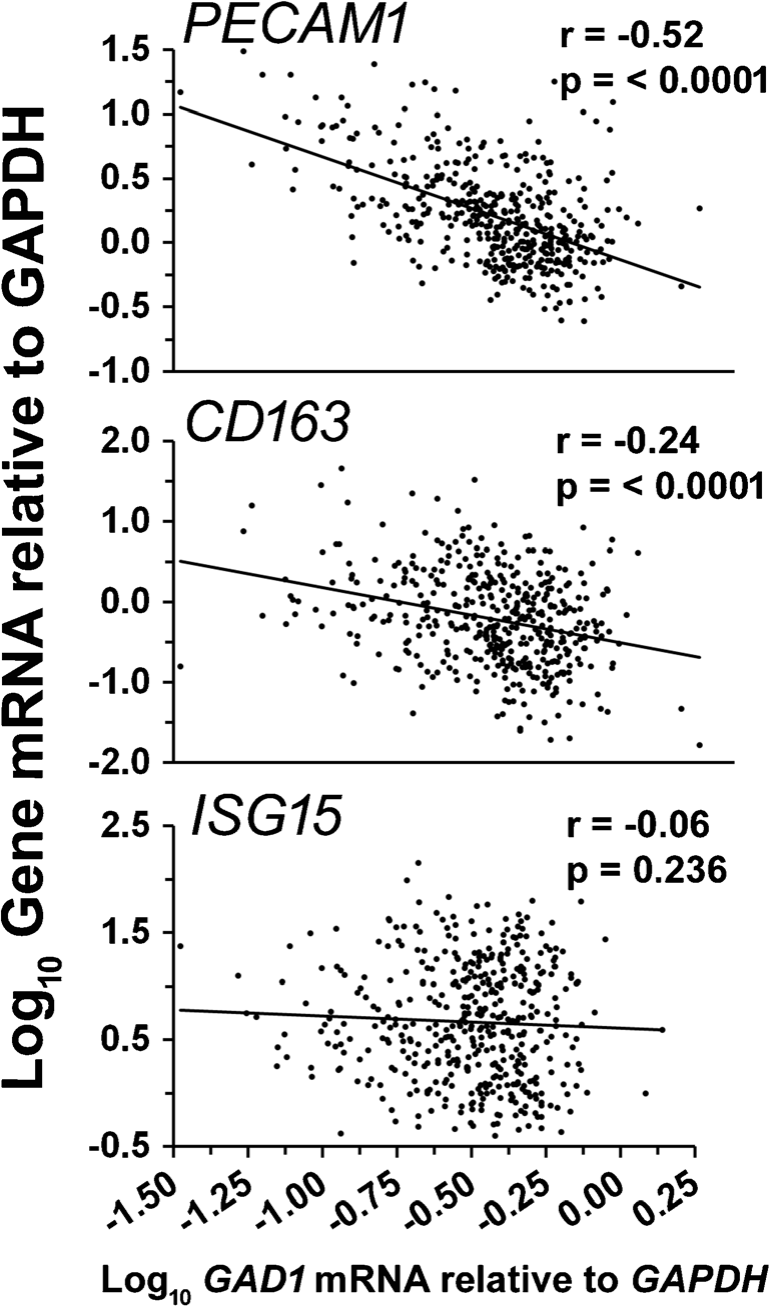
A marker of increased endothelial activation (*PECAM1* mRNA; CD31) is correlated strongly with abnormally low GABAergic transmission in brain specimens from HIV-infected people (top panel). Four hundred forty-nine infected patients were assayed for glutamic acid decarboxylase (67 kDa) gene expression in frontal neocortex (*GAD1* mRNA), which is the rate-limiting step in GABA synthesis. *GAD1* correlations with a prototypal viral inflammatory interferon response marker (*ISG15* mRNA; bottom panel) and a prototypal macrophage marker of brain HIV infection (*CD163* mRNA; middle panel) are not as strong as the endothelial cell marker. The regression line for the endothelial cell marker has the steepest slope, and the correlations have statistically different slopes from each other (*r* to *Z* transformation; *p* < 0.01)

Neurochemical genes related to synaptic transmission also are regulated on brain gene arrays, and many aspects have undergone solid confirmation using 449 HIV-infected brain specimens (Table 1). One example is that dopamine type 2 receptor long isoform (*DRD2*) expression is dysregulated, possibly in response to heightened presynaptic tone (Gelman et al. 2012a, b). Neuronal genes and proteins associated with the neurotransmitter GABA are downregulated in the apparent absence of any dropout of inhibitory neurons (Buzhdygan et al. 2016). The loss of GABAergic transmission correlates most strongly with the increase in endothelial activation markers. In contrast, brain markers that drive HIV replication, or respond to it, such as *CD163* and *ISG15* mRNAs, respectively (Kim et al. 2006; Okumura et al. 2006), are less strongly correlated with the GABAergic disturbance (Fig. 1). The fact that these neurochemical changes do not result from HIVE, when weighed against prior dogma in the field, can give the impression of being antithetical. The divergence of these markers away from HIVE is, in fact, what needs to be imitated in models of HAND in virally suppressed patients.

**Table 1:** Neurochemical abnormalities in 449 HIV-infected patients

Characteristic	Neurotransmitter system			
	Dopamine (*DRD2L*)	Enkephalin (*PENK*)	GABA (*GAD1*)	Serotonin (*HTR2C*)
Nature of change in HIV/AIDS	“Abnormally” low	Abnormally low	Abnormally low	Abnormally low
Postulated synaptic physiology	Heightened presynaptic tone; dampened postsynaptic tone	Dampened presynaptic tone	Dampened pre- and postsynaptic tone	Dampened postsynaptic tone
Association with HAND	Association with “No HAND”; (“abnormal” is beneficial)	No known circuit	Yes in cingulate, no in frontal cortex	Limited to verbal fluency testing in frontal circuit
Associated with HIVE	Association with “No HIVE”	No	No	No
Worsens neuropsychology?	Yes if high	Unknown	Yes in cingulate, not in frontal cortex	Specifically verbal fluency
Linkage to endothelial activation markers?	No	Yes	Yes	Yes

At least four neurotransmitter systems are abnormal in HIV-infected people without encephalitis. mRNA of frontal cortex is portrayed in this table. From Gelman et al. (2012a, b) and Buzhdygan et al. (2016)

## One hit versus multiple hit concepts for HAND pathophysiology

**Fig. 2:**
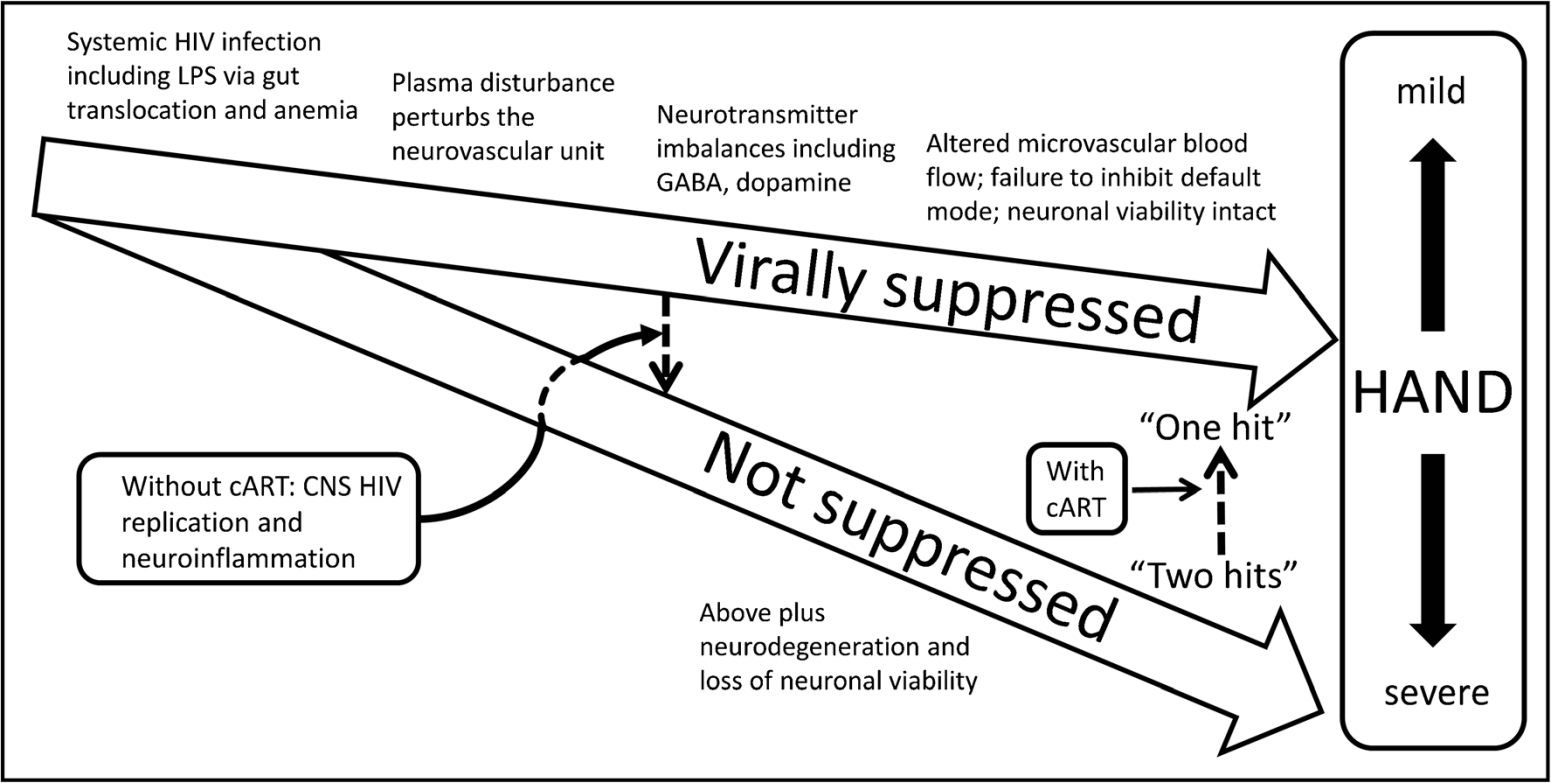
In the clinic, HIV neurocognitive disorders (HAND) can appear to be one pathophysiological process on a continuum of various intensities (box at right). It is likely that multiple pathophysiologies contribute to the range of clinical intensity. A simplified “multi-hit” hypothesis addresses observations of patients with differing severities of HAND. The suggested schema shows one major hit that occurs in patients not virally suppressed. This hit results from CNS viral replication, CNS inflammation, and possibly neurodegeneration and produces severe neurocognitive impairment. Another type of hit persists in virally suppressed patients that leads to milder forms of HAND. Systemic changes due to smoldering HIV infection may drive the latter hit, and the neurovascular unit transmits systemic anomalies from blood to brain. Other potential hits that modify the intensity of the clinical picture can include substance abuse, systemic or CNS pathogens, and the aging process itself. Diverse pathophysiological changes converge to produce a clinical phenotype of variable clinical severity. Experimental models of HAND should establish precisely which pathophysiological process is imitated and exactly which patient population is emulated in the model

Mild HAND with viral suppression, versus severe HAND without it, might appear clinically to be one thing, differing only in intensity and various comorbid clinical settings. The neuropathological “gap” as discussed above suggests very strongly that HAND is a clinically defined nosology that involves multiple converging mechanisms. The continuum of changes as one progresses from asymptomatic neurocognitive impairment (ANI), to mild neurocognitive disorder (MND), to HIV-associated dementia (HAD) is not likely to represent different intensities of a unitary pathophysiology. Figure 2 diagrams a simplified “multi-hit” model of HAND that addresses some observations in HIV-infected people with and without viral suppression. A substantial “hit” occurred in patients not virally suppressed and resulted from CNS viral replication, CNS inflammation, and possibly neurodegeneration. This scenario can produce severe neurocognitive impairment and HAD. Another subtler type of hit is unmasked in virally suppressed patients and leads to milder forms of HAND. Systemic changes due to smoldering HIV infection and the “undercurrent” of chronic systemic disease may drive the latter hit (Carroll and Brew 2017; Manji et al. 2013; Spudich et al. 2011). In the scenario suggested in Fig. 2, the focal point that mediates systemic disease and brain dysfunction could be the neurovascular unit. Another potential “hit” is the postulated “legacy effect,” from CNS damage that could occur before the patient becomes symptomatic (Simioni et al. 2010; Tan and McArthur 2012; Brew 2010). An early and lasting “hit” is suggested to take place shortly after infection due to transient aseptic meningitis, as suggested in brain images obtained just after initial infection with HIV (Ragin et al. 2015). Other potential hits that modify the intensity of the neuropsychological picture include substance abuse, intercurrent systemic or CNS infection, and the aging process itself (Shuster and Gonzalez 2012; Gill and Kolson 2014; Chen et al. 2014). Mechanistically diverse insults accumulate and converge to produce impairment of variable intensity. Experimental models of HAND should strive to define precisely which pathophysiological process that occurs in virally suppressed patients is emulated using model systems. At present, the use of models in NeuroAIDS literature, and the clinical nosology itself, tend to imply that there is a unitary mechanism in play, which applies generally to HAND. In the future, the literature needs to specify what type of “hit” the study aims to emulate. Specifically, the mechanism accepted for patients with HIVE cannot be treated as an “all purpose” pathophysiology for ANI, MND, HAD, and HAND generally.

## Models of viral latency in the CNS of humans

Determining the scientific foundation of viral latency/reservoirs in the CNS and throughout the human body is a formidable challenge (Marban et al. 2016). Establishing working models of viral latency/reservoirs that emulate “the real thing” looms as a critical and potentially daunting milestone. One key question is whether clinically relevant challenges in the HAND and latency/reservoir fields overlap, versus being primarily non-convergent problems. If the latter is true, disease modeling of the two problems should diverge as well. The diminishing connection between brain HIV RNA burden and HAND suggests that the pool of latent HIV DNA in the brain also is unlikely to drive neurocognitive impairment. As well, the therapeutic goal of treating HAND (to improve brain function) differs sharply from the goal of eradicating latent/reservoir HIV in the CNS (to cure HIV/AIDS). It follows that HAND and viral latency/reservoirs represent distinct fields of study that will require specialized research tools and separate models (Gelman 2015).

HIV DNA can be detected biochemically in most brains examined from people who have undergone viral suppression (Gelman et al. 2013; Lamers et al. 2016). The HIV DNA data from human brain specimens imply that a CNS HIV DNA reservoir is indeed present. Some evidence in model systems implies that the CNS HIV DNA pool is capable of reseeding the replicating pool (Gama et al. 2017).There remains room to question the interpretation obtained thus far from autopsy data. First, studies of the systemic HIV reservoir show that the bulk of the infected body’s HIV DNA is replication-defective (Bruner et al. 2016; Henrich et al. 2017). Second, phagocytosis of HIV-infected brain cells by macrophages or astrocytes could be an important barrier to establishing authentic HIV infection (Baxter et al. 2014; Calantone et al. 2014; DiNapoli et al. 2017; Russell et al. 2017). Finally, ART is routinely stopped in terminal health care; without it, transcriptionally competent HIV DNA may not be truly latent at the time of autopsy, even if virus was suppressed at the prior clinic visit. In total, these observations raise the possibility that “latent” CNS HIV DNA could be a compartmentalized “dead end” that cannot sustain HIV infection, and it may not be truly latent when quantified in a patient that stopped taking cART.

Another key question pertains to brain cells that maintain the latent HIV DNA pool. In human lymphoid tissue and blood, the latent viral pool resides primarily in T lymphocytes phenotypically typed as central memory or transitional memory cells. That is far less likely to be true in nonlymphoid organs that contain few lymphocytes. In the CNS, infected cells are CD163+ mononuclear phagocytes, which suggests a myeloid (macrophage) or a yolk sac (microglial cell)-derived reservoir, versus lymphocytes (Kim et al. 2006; Le Douce et al. 2010). HIV-infected resident histiocytes in the brain and other non-lymphoid organs, such as alveolar macrophages in the lungs, are likely to host latent pools in some and perhaps most body compartments. We know that replicating HIV in the brain occurs primarily in M2 macrophages that are CD163+ and CD16+ (Fischer-Smith et al. 2008); it remains to be determined whether a subset of this particular macrophage phenotype is what harbors latent HIV DNA in the CNS. These cells are extremely difficult to find in the CNS of virally suppressed patients, who generally have far less than 500,000 copies of HIV DNA in the entire brain specimen (not illustrated).

**Fig. 3:**
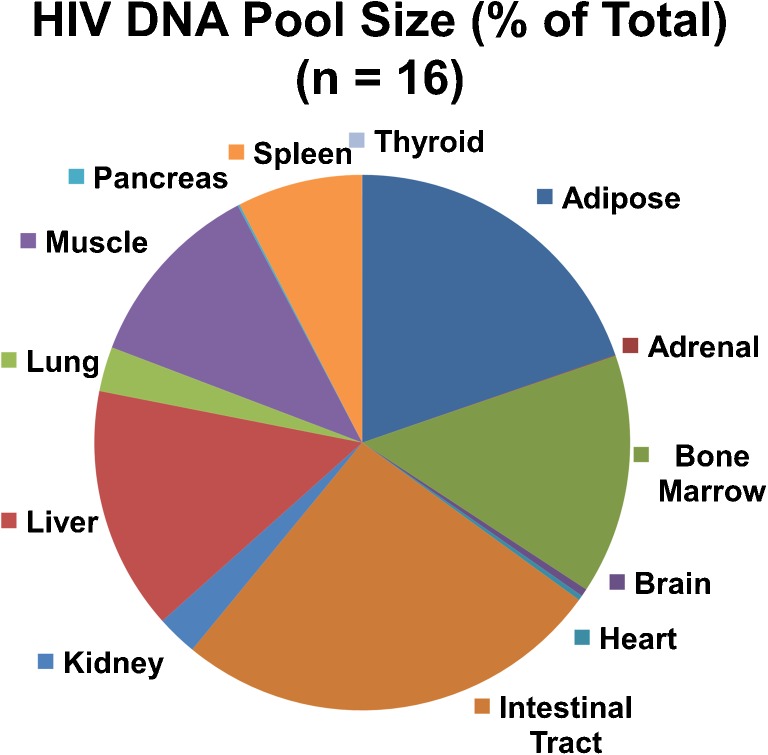
The pie chart illustrates sizes of HIV DNA pools in human organs and body compartments. Pool sizes were determined by measuring the concentration of the HIV DNA and the mass of the compartment. Pool sizes were corrected for HIV DNA due to blood pooling in the organs. The HIV DNA pool size in the brain is relatively small in comparison to other organs. Note that a compartment such as muscle contains a relatively low concentration of HIV DNA, but the pool size is large nevertheless because the compartment is massive in size

**Fig. 4:**
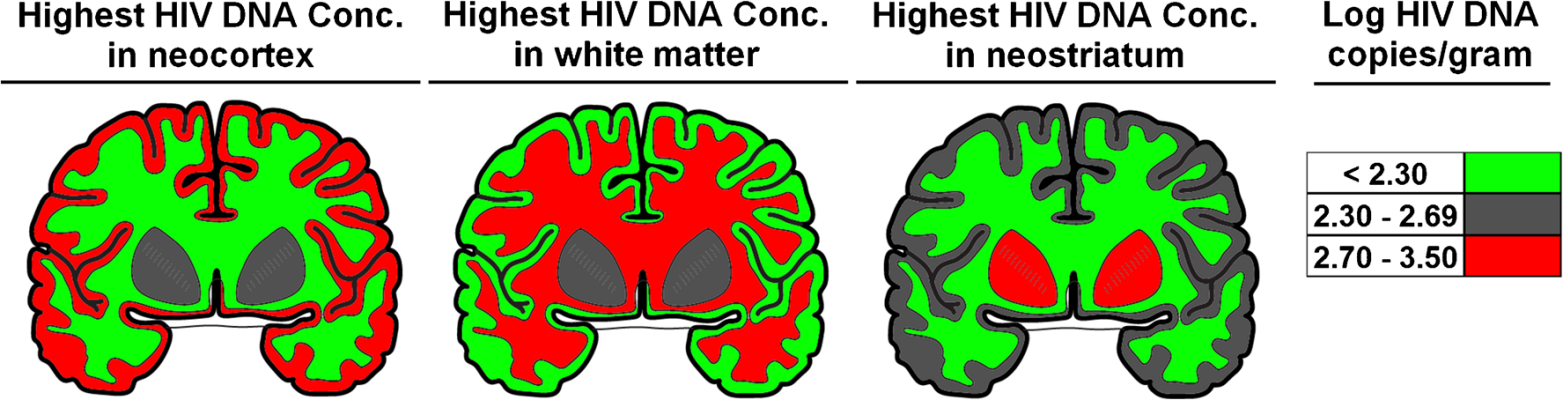
Concentrations of HIV DNA in the brain of three infected patients. The brain specimen at the left has the highest concentration of HIV DNA in neocortex. The middle specimen has highest concentration in white matter. The specimen at the right has the highest concentration in the neostriatum. Measurement of 29 human brain specimens showed that almost no patient’s brain distribution of HIV DNA conforms to the average of the group. Differing classes of patients exist with regard to the CNS distribution of the HIV DNA reservoir. Reasons for the wide variation of the CNS distribution of HIV DNA in the human population remain to be determined

We embarked recently on the initial characterization of the HIV DNA in deep body compartments of autopsy specimens. In whole-body maps corrected for blood transiting through organs, we find that the HIV DNA pool in the CNS is small relative to the total body pool (Fig. 3). The duration of HIV infection (years) has no apparent influence on the size of the HIV DNA pool in the brain, which does vary substantially. We also found no stereotyped regional pattern of HIV DNA in the brain. Some have a higher concentration in gray matter compartments relative to white matter; others do not (Fig. 4). Also important was that the relationship between viral replication in blood plasma versus the size of the HIV DNA pool in the brain showed an apparent threshold. The brain HIV DNA pool size begins to expand when systemic viral replication increases above about 30,000 copies of HIV RNA per ml in plasma. As plasma replication is suppressed to levels below about 30,000 copies, the brain HIV DNA pool size does not decrease further. In longitudinal studies spanning up to 12 years of sample collection, we observed that patients who discontinue ART and resume active viral replication do not have a substantial increase of HIV DNA in peripheral blood mononuclear cells (PBMCs); when cART was restarted and replication was suppressed, their HIV DNA in PBMCs did not decrease (not shown). At the present time, *intense* viral suppression is not a very strong determinant of the size of the latent HIV DNA pool in some and perhaps all body compartments, including the brain and PBMCs (Siliciano and Siliciano 2015). In the future, when medicines that reduce HIV DNA pools become available, the intensity of viral suppression could become a more important influence on the size of the HIV DNA pools in the body.

## Faithfulness of NeuroAIDS models to “the real thing”

Human brain specimens and images inform us regarding how to heighten the clinical relevancy of experimental models of HAND and CNS HIV latency in virally suppressed people. In order to faithfully imitate “the real thing,” models of HAND and CNS viral latency need to evolve and adapt to shifting clinical scenarios. Given the available evidence from human brain specimens, some general guidelines for model selection and applications to virally suppressed patients are implied:

1. Mechanisms that apply to virally suppressed patients given ART are different from unsuppressed patients. When assigning the clinical relevancy of experimental designs and models, the differences need to be highlighted and not obscured. Investigators should identify precisely what aspect of the problem that the model being used tries to emulate. Suggesting that the pathophysiology of HAND in unsuppressed patients with HIVE is relevant to virally suppressed patients with HAND is out-of-date and probably erroneous.
2. A single model does not faithfully emulate all aspects of clinically relevant CNS disease. Pathophysiologies of ANI, MCD, and HAD are not necessarily identical because the neuropathology for each is unclear. Comorbid conditions vary across infected patient populations. Non-progressive “legacy” effects of HIV infection are temporally and mechanistically different than progressively worsening changes.
3. Appropriate models of HAND differ from those that are useful to study viral latency and eradication in the CNS. The goal of latency models is to determine how to eliminate latent viral DNA; the goal of HAND models is to determine how to restore neuropsychological function. Models can address one or the other and seldom if ever both.
4. Promulgating awareness and respect for the limitations of our models is as important as touting their strengths. A conscientious researcher should ask and answer critical questions: “What aspects of this model match observations that have been made in virally suppressed humans? What aspects of the model are unlikely to be applicable to such patients?”
